# Caught in the act: the invasion of a viral vector changes viral prevalence and titre in native honeybees and bumblebees

**DOI:** 10.1098/rsbl.2023.0600

**Published:** 2024-05-08

**Authors:** Jana Dobelmann, Robyn Manley, Lena Wilfert

**Affiliations:** ^1^ Institute of Evolutionary Ecology and Conservation Genomics, University of Ulm, Albert-Einstein-Allee 11, Ulm 89081, Germany; ^2^ University of Exeter, Exeter EX4 4QD, UK

**Keywords:** virus transmission, *Varroa*, deformed wing virus, honeybee

## Abstract

Novel transmission routes change pathogen landscapes and may facilitate disease emergence. The varroa mite is a virus vector that switched to western honeybees at the beginning of the last century, leading to hive mortality, particularly in combination with RNA viruses. A recent invasion of varroa on the French island of Ushant introduced vector-mediated transmission to one of the last varroa-naive native honeybee populations and caused rapid changes in the honeybee viral community. These changes were characterized by a drastic increase in deformed wing virus type B prevalence and titre in honeybees, as well as knock-on effects in bumblebees, particularly in the year following the invasion. Slow bee paralysis virus also appeared in honeybees and bumblebees, with a 1 year delay, while black queen cell virus declined in honeybees. This study highlights the rapid and far-reaching effects of vector-borne transmission that can extend beyond the directly affected host species, and that the direction of the effect depends on the pathogen’s virulence.

## 1. Introduction

Emerging diseases pose a threat not only to humans, livestock and crops but also to wildlife populations, as exemplified by the spread of diseases such as mycoplasma in American bird populations [1] or the global spread of chytridiomycosis in amphibians [2]. Disease emergence can be facilitated by changes in host–parasite ecology [3], such as changes in transmission routes. For instance, the acquisition of airborne transmission was key to all four influenza pandemics in the past century [4]. Vector-borne transmission plays a special role; with the acquisition of vector-borne transmission, pathogens can evade many of the hosts’ innate immune defences, bypassing the body wall, while often simultaneously increasing inoculum size [5]. Under dose dependency, a larger inoculum can increase infection intensity and prevalence and may, ultimately, increase virulence [6]. Acquiring vector-mediated transmission is thus expected to reduce host population health while increasing pathogen fitness.

The acquisition of vector-borne transmission is a rare evolutionary event, limiting opportunities to study the evolutionary and ecological impacts of this crucial transmission route. The honeybee *Apis mellifera* and its RNA viruses present a rare exception: at the beginning of the last century, *Varroa destructor* (hereafter varroa) switched from its native host the eastern honeybee (*Apis cerana*) to the western honeybee (*A. mellifera*) [7,8]. Its global spread was followed by increased hive mortality [7], which has been largely attributed to this ectoparasite vectoring RNA viruses. Varroa transmits viral particles directly into the bee’s body cavity [9] while feeding on the fat body [10] and removing haemolymph [11]. The key culprit in varroa-associated increased hive mortalities has been identified as the RNA virus deformed wing virus (DWV). Varroa dramatically increases DWV prevalence and titre [12,13]. Numerous field studies have shown that the combination of varroa and DWV, previously considered largely avirulent, are the main drivers of high over-winter mortality rates that have plagued beekeepers in temperate regions [14–17].

The re-emergence of DWV and its global spread followed varroa’s host switch to western honeybees [8,18]. In addition, there are multiple other honeybee viral pathogens that are vectored by varroa [19] and that have also been shown to increase in prevalence in the presence of this vector, such as acute bee paralysis virus (ABPV), black queen cell virus (BQCV) and slow bee paralysis virus (SBPV) [19–23]. Although varroa uniquely parasitizes honeybees, its effect as a vector is not limited to honeybees. Instead, it indirectly increases viral prevalence and infection intensity in other species, such as wasps that prey on bees [24] and wild bumblebees that share floral resources [20,25,26].

The acquisition of varroa in the western honeybee has introduced vector-borne transmission into a complex host–pathogen system, where it was previously absent. This allows studying how vector-borne transmission affects the evolution and epidemiology of such systems, such as how viral populations change with vector-borne transmission [8,27], how viral responses depend on their virulence [28] and how communities are affected [20,25]. Studying invasions [21,27] and contrasting invaded areas with varroa-free refugia [20,25,29] has been particularly fruitful. However, as varroa is reaching global distribution, opportunities to study its invasion are becoming rare [30], particularly in regions where *A. mellifera*, native to Europe and Africa, is part of a co-evolved host–pathogen community. A small number of islands in the English Channel (Scilly Isles and Alderney) and the Irish Sea (Isle of Man) remain the last varroa-naive populations in the western honeybees’ temperate native range [25]. Until recently, the small French Island of Ushant was one of these rare native refugia. However, in June 2021, beekeepers on Ushant first reported mites and an alarming 50–70% hive mortality over the season [31]. Although no increased over-winter mortality was observed in the year preceding the varroa detection, the heavy varroa infestation indicated that the introduction could have gone unnoticed for a while, with a likely introduction in 2020 [31]. To directly study the effect of vector acquisition on the native, co-evolved host–pathogen community of *A. mellifera* and associated wild bumblebees, we compare viral prevalence and titres in honeybees and wild bumblebees on Ushant before and right after the introduction of varroa in this study.

## 2. Material and methods

### (a) Sampling and RNA extraction

Foraging bees (*A. mellifera, Bombus terrestris* and *B. pascuorum*) were collected from flowers across the entire area of Ushant, France, in late June and early July 2015 (pre-varroa), and 2021 and 2022 (post-varroa; details in electronic supplementary material, M1). Samples from 2015 were collected by Manley *et al*. [25]. Bees were frozen within 24 h of collection and transported to the lab in a dry shipper. *B. terrestris* was distinguished from *B. lucorum* by PCR with species-specific length polymorphism (details in electronic supplementary material, M2). Following methods described in Manley *et al.* [25], tissue from half bees (laterally bisected) was homogenized (speed 5 m s^−1^ for 25 s with three cycles and 20 s pause using a FisherBrand Bead Mill 24) and RNA was extracted using 1.3 ml TRI-Reagent® (Sigma-Aldrich) and 0.1 ml bromo-chloro-propanol (Sigma-Aldrich). RNA was eluted in 80, 100 or 120 µl H_2_O, for *A. mellifera, B. pascuorum* and *B. terrestris,* respectively. RNA concentrations (including 2015 samples) were measured by fluorescent dye (QuantiFluor® RNA System, Promega). In total, we included 257 *A. mellifera* (30 in 2015, 159 in 2021, 68 in 2022), 117 *B. pascuorum* (28 in 2015, 35 in 2021, 54 in 2022) and 138 *B. terrestris* (13 in 2015, 60 in 2021, 65 in 2022).

### (b) Viral prevalence screening

Prevalence data for 2015 were taken from Manley *et al.* [20,25]. For 2021 and 2022, we followed the methods described there. Briefly, 600–3750 ng RNA was reverse transcribed using random hexamers and GoScript™ reverse transcriptase (Promega) according to the manufacturer’s instructions to screen for common honeybee pathogens (DWV type A and B [32], SBPV and BQCV). PCR was performed using GoTaq® Flexi (Promega) with 2.5 mM MgCl_2_, 0.2 mM dNTPs, 0.5 µM each primer, 0.375 U polymerase (0.75 U for SBPV) and 2 µl 1 : 10 diluted cDNA in 15 µl reactions (primer sequences and cycling protocols in electronic supplementary material, table S1). Every run included a known virus-positive sample and water as a negative control. Five microlitres of PCR product was visualized by 1.5% TAE agarose gel electrophoresis and staining with RedSafe^TM^ (Intron Biotechnology) using HyperLadder™ 50 bp (Bioline) to check the fragment size. Prevalence was determined by examining the presence of a distinct band on a gel.

### (c) Virus quantification

DWV virus titres were quantified in all DWV-positive samples from 2015 and 10 randomly selected positive samples per year and species from 2021 and 2022. qPCR standards for DWV-A and DWV-B were created from an eight-point serial dilution (1 : 10) of *in vitro* transcribed RNA generated by cloning PCR products into a plasmid (electronic supplementary material, M3). Four hundred nanograms template RNA or 4 × 10^1^ – 4 × 10^−6^ ng DWV standard were reverse transcribed using random hexamers with GoScript™ reverse transcriptase (Promega) and diluted 1 : 10. Reactions were performed on a qTower (Analytics Jena) or QuantStudio5 (Applied Biosystems) machine with GoTaq® qPCR kit (Promega) in duplicates using 4 µl template and including negative controls, standards and internal reference genes to check host cDNA concentrations (primer sequences and efficiency in electronic supplementary material, table S2). The quality of each reaction was assessed by reviewing the amplification plot and melting curve. As all samples were tested positive before, the Ct threshold was set to 40 and reactions were repeated when inconclusive. We estimated the amount of DWV in 400 ng RNA by interpolating the sample Ct value to the standard curve. Copy number was determined using the formula: copy number = cDNA concentration (ng) × 6.02 × 10^23^ (copies mol^−1^)/length (bp) × 6.6 × 10^11^ (ng mol^−1^). Using the total amount of extracted RNA from half bees, we then calculated the total number of viral copies per bee.

### (d) Statistical analyses

Statistical analyses were performed in R v. 4.2.0 [33]. True pathogen prevalence with 98% sensitivity (true-positive rate) and specificity (true-negative rate) was calculated using the *epiR* package [34]. To assess if the proportion of infected versus uninfected bees differed between sampling years and between bee species, we used Fisher’s exact test in pairwise comparisons. For virus quantification, estimates of viral copy numbers per bee were log10 transformed for statistical analyses. Wilcoxon rank sum tests using the package *ggpubr* v. 0.6.0 [35] were performed to test whether viral loads in honeybees and bumblebees changed between years. To correct for multiple testing, we used the false-discovery rate (*Q* = 0.05). No significant results were rejected under false-discovery rate correction and unadjusted *p*-values are reported.

## 3. Results

### (a) Viral prevalence in honey and bumblebees

**Figure 1.**
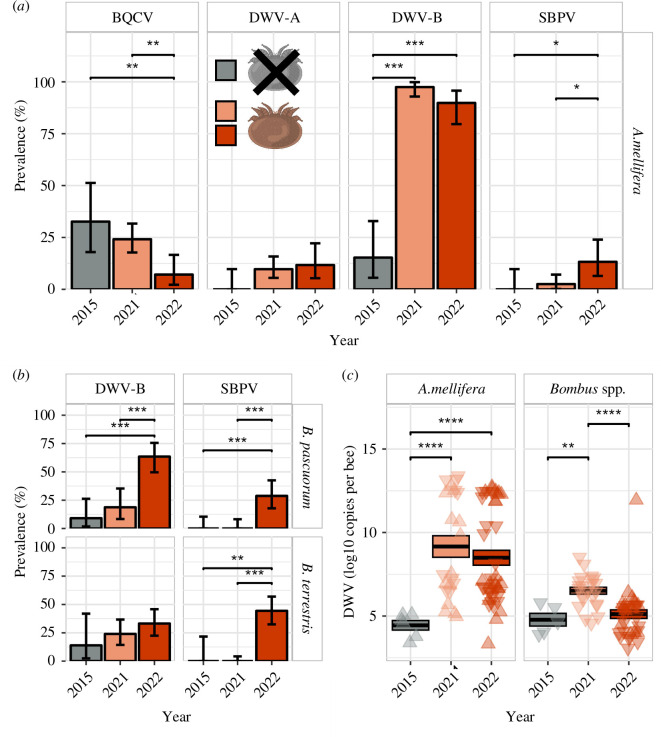
Viral prevalence and titres in honeybees (*Apis mellifera*) and bumblebees (*Bombus pascuorum* and *B. terrestris*) before (grey) and after (orange and red) varroa invaded the island of Ushant in France. Significantly different prevalences or titres are indicated by * for *p* < 0.05, ** for *p* < 0.01, *** for *p* < 0.001 and **** for *p* < 0.0001. (***a***) Black queen cell virus (BQCV), deformed wing virus type A (DWV-A) and type B (DWV-B) and slow bee paralysis virus (SBPV) prevalence in *A. mellifera*. Bars show true prevalence with error bars indicating 95% confidence intervals. Actual prevalence was used when all samples tested negative. (***b***) DWV-B and SBPV prevalence in *Bombus* spp. BQCV and DWV-A were not detected. Bars show true prevalence with error bars indicating 95% confidence intervals. Actual prevalence was used when all samples tested negative. (***c***) DWV titres in *A. mellifera* and *Bombus* spp. Only samples tested positive by endpoint PCR were tested, which may lead to inflated copy numbers. Up-pointing triangles show DWV-A and down-pointing triangles show DWV-B. Bars show mean values and boxes indicate standard error.

DWV-B significantly increased in *A. mellifera* from 15.28% in 2015 to 97.50% in 2021 and 89.83% in 2022 (Fisher’s exact test: both *p* < 0.001, figure 1*a*). DWV-A was not detected in 2015 but was found at low prevalence (around 10%) in 2021 and 2022 post varroa. In bumblebees, DWV-B prevalence also increased after varroa arrived on the island (2015: 9.08% and 13.94%; 2021: 18.75% and 23.96%; 2022: 63.50% and 33.17% in *B. pascuorum* and *B. terrestris*, respectively). In *B. pascuorum*, DWV-B prevalence in 2022 was significantly higher than in 2015 and in 2021 (both *p* < 0.001; figure 1*b*). Increases in *B. terrestris* were not significant (all *p *> 0.05), however, only 13 *B. terrestris* were available from 2015 (pre-varroa). DWV-A was not detected in bumblebees. In 2015, DWV-B prevalence did not differ between the three species (all *p* > 0.05). Yet, after varroa invaded, prevalences in 2021 and 2022 were significantly higher in *A. mellifera* than in *B. pascuorum* and *B. terrestris* (2015 versus 2021: both *p* < 0.001, 2015 versus 2022: *p* = 0.001 and *p* > 0.001, respectively). *B. pascuorum* thereby had a higher prevalence than *B. terrestris* in 2022 (*p* = 0.017)

SBPV also increased in all species: SBPV was absent in 2015 and then appeared post-varroa invasion in 2021 in *A. mellifera* at low prevalence (2.50 %, *p* = 0.599) and in 2022 in all three species (increase in 2015 versus 2022: *p* < 0.001 and *p* = 0.001 in *B. pascuorum* and *B. terrestris*, respectively, and in 2021 versus 2022: *p* < 0.001 in both *Bombus* spp., in *A. mellifera p* = 0.012, figure 1*a* and *b*). SBPV prevalence in 2022 tended to be higher in bumblebees (*B. pascuorum*: 28.78% and *B. terrestris*: 44.39 %) compared with honeybees (13.24 %, *p* = 0.074 and *p* < 0.001, respectively). BQCV was only detected in honeybees. In contrast to other viruses, its prevalence decreased over time from 32.64% in 2015 and 24.12% in 2021 to only 7.11% in 2022 (both: *p* = 0.006, decrease from 2015 to 2021: *p* = 0371, figure 1*a*).

### (b) Viral titres in honey and bumblebees

Before varroa introduced vector-mediated viral transmission on Ushant, only 10 individuals (five *A. mellifera*, two *B. pascuorum* and three *B. terrestris*) out of 71 samples tested positive for DWV [25]. Viral titres were on average 10^5^ copies per bee in all species (figure 1*c*). These titres drastically increased to an average of 10^10^ copies in *A. mellifera* and 10^7^ in *Bombus* spp. after varroa arrived in 2021. Infection intensity remained high in *A. mellifera* in 2022 (10^10^ viral copies per bee) but decreased to 10^6^ copies in *Bombus* spp. Copy numbers in honeybees were significantly increased from pre- to post-varroa samples (Wilcoxon rank test: 2015 versus 2021: *p* < 0.001; 2015 versus 2022: *p* < 0.001). No difference was found between years after varroa was found on the island (Wilcoxon rank test, 2021 versus 2022: *p* = 0.394). In bumblebees, viral loads were significantly lower in 2015 than in 2021 (Wilcoxon rank tests, *p* = 0.007) and in 2022 (*p* = 0.041). DWV titres in 2021 were also higher than in 2022 (*p* < 0.001).

## 4. Discussion

The varroa invasion on Ushant was followed by rapid changes in the viral community of *A. mellifera*, as well as knock-on effects in the associated host community, represented by two wild bumblebee species. These changes were characterized by a drastic increase in DWV-B prevalence and titre in honeybees, followed by increases in bumblebees particularly in the year following the varroa detection; similarly, SBPV appeared first in honeybees and then with a 1 year delay in the bumblebees. In contrast, BQCV declined in honeybees and was not detected in bumblebees. This highlights the rapid and drastic effects of vector-borne transmission that may reach beyond the directly affected host species, and that the direction of the effect may depend on the pathogen’s virulence and reservoir species.

In varroa-free populations, common honeybee pathogens, including DWV, frequently occur at low prevalence with low viral titres [21,23,25,27,36] or are mostly absent, such as in Australia [29]. The rapid and dramatic increase in DWV frequency and titre in the honeybee’s native community following the Ushant invasion highlights the importance of varroa control and mirrors the effects observed in non-native populations in New Zealand [21] and Hawai’i [27], and fits with the long-term pattern observed in European islands [25]. It should nevertheless be noted that we cannot fully exclude other factors, such as climate change, playing a role in this change. In bumblebees, which do not have contact with the vector, we observed a delayed increase in prevalence, suggesting that the knock-on effects of vector-borne transmission in this multi-host–pathogen system only take hold over several infection cycles. The increase in viral titre to an average of 10^10^ viral copies per bee in infected honeybees and 10^7^ in bumblebees was immediate and also concurs with the long-term pattern described by Manley *et al.* [25].

DWV is a multi-strain pathogen [32]. The post-vector-invasion DWV-epidemic in New Zealand and Hawai’i consisted of DWV-A [21,27]. While this variant globally spread in parallel with varroa over the last century [8,18], the recently emerged DWV-B [25] has rapidly gained in prevalence in the UK and the US, potentially replacing the older DWV-A [37]. For instance, in the US, DWV-B increased from 3% prevalence in 2010 to 66% in 2016 [38]. The higher DWV-B prevalence observed in 2021 and 2022 on Ushant follows this pattern, yet there seems to be no complete strain replacement as DWV-A prevalence increased too, albeit non-significantly and at a low level. DWV-A could have been rare in 2015 so it remained undetected. Experimentally, DWV-B has been shown to have a competitive advantage over DWV-A irrespective of varroa presence [39,40]. There is growing evidence of active replication of DWV-A and DWV-B in varroa [41–43] although vector–virus interactions differ between genotypes [ 44]. Wild bees frequently show a high prevalence of DWV but lower titres than honeybees [45]. DWV can replicate in bumblebees [46], but as viral transmission between bumblebees may be inefficient [47], bumblebees may mostly get infected by spillover from honeybees. Furthermore, in contrast to honeybees, the effects of DWV infection on bumblebees are unclear [26,46,48].

SBPV increased in all three surveyed species but with a delay of one season compared with DWV. Manley *et al.* [20] found that under long-term exposure, SBPV had the highest prevalence in the varroa-invaded islands, higher both than in refugia islands and in the invaded mainland, suggesting an interaction between island populations and vector-borne transmission. When infections co-occur with other stressors, SBPV is virulent in bumblebees [49].

In contrast to Mondet *et al.* [21], who found BQCV to increase after the invasion, we found overall low BQCV prevalence and a decrease. This pattern may be caused by BQCV’s relatively high virulence: highly virulent viruses may kill their host too fast for the vector to complete its reproductive cycle, resulting in a negative association between the virus and vector [50]. Remnant *et al.* [28] have shown that pupal injection of BQCV leads to premature death and suboptimal transmission, and potentially a negative association with varroa. However, in colonies with experimentally increased or lowered varroa infestation levels, BQCV prevalence did not differ [39]. Varroa-free bees in Australia tolerate high BQCV prevalence, which coincides with BQCV being the most common ‘honeybee’ pathogen in Australian wild pollinators [51]. The decrease in BQCV prevalence and increase in DWV prevalence and virulence upon vector invasion found here suggests that viral virulence may play a role in predicting the outcome of acquiring vector-borne transmission, as the evolutionary trade-off between virulence and transmission can change with novel transmission routes [52]. Vector-mediated changes in pathogen landscapes could thus both lead to the emergence of pathogens and to their decline—in prevalence or eventually virulence—of previously important pathogens.

Exploiting species invasions as natural experiments to examine pathogen dynamics ahead and behind an invasion front allows us to link theoretical, empirical and experimental studies [53]. The varroa invasion on Ushant provides a rare example where longitudinal data that covered the arrival of a vector shed light on how changes in transmission routes can quickly affect pathogen dynamics. As disease emergence probability rises with environmental change [54], understanding the role of vectors in pathogen transmission is crucial for assessing and managing the impact that vector transmission has on pathogen communities.

## Data Availability

The datasets supporting this article have been uploaded as part of the electronic supplementary material [55].
